# A Child With Concomitant Neuroblastoma and IgA Nephropathy: A Case Report and Literature Review

**DOI:** 10.7759/cureus.60089

**Published:** 2024-05-11

**Authors:** Ling Yu, Jingjing Wang, Chunyue Feng, Guoping Huang, Weizhong Gu, Jieni Xiong, Jianhua Mao

**Affiliations:** 1 Department of Nephrology, Children’s Hospital, Zhejiang University School of Medicine, Hangzhou, CHN; 2 Department of Pathology, Children’s Hospital, Zhejiang University School of Medicine, Hangzhou, CHN; 3 Department of Surgical Oncology, Children’s Hospital, Zhejiang University School of Medicine, Hangzhou, CHN

**Keywords:** proteinuria, paraneoplastic condition, child, neuroblastoma, iga nephropathy

## Abstract

Concurrent malignancy and IgA nephropathy are rare. Despite the lack of solid experimental evidence, there are theoretical hypotheses of pathophysiology for the development of glomerular damage in cancer patients, like aberrant immune activities. Here, we describe a nine-year-old child who was admitted due to nephrotic syndrome. Abdominal imaging examination accidentally revealed a retroperitoneal tumor, and surgical resection was performed with a pathological diagnosis of neuroblastoma. However, complete removal of the tumor had no impact on the clinical manifestation of nephrotic syndrome, like proteinuria. The use of corticosteroids alone only led to a partial resolution of proteinuria, and resistance developed after one month of treatment. A further kidney biopsy was performed, which suggested IgA nephropathy. Clinical remission of IgA nephropathy was achieved after standard combination treatment of corticosteroids and mycophenolate mofetil for 10 months. This study represented the first case report of neuroblastoma associated with IgA nephropathy. We postulated that IgA nephropathy pathogenesis might be associated with neuroblastoma, though a coincidence of these two conditions cannot be fully excluded. Standard treatment for IgA nephropathy is applicable for patients with concomitant cancer.

## Introduction

Immunoglobulin-A (IgA) nephropathy is the most prevalent primary glomerulonephritis worldwide [1]. The etiology of IgA nephropathy remains unclear. A concomitant malignancy with IgA nephropathy has been reported and represents a rare clinical manifestation [2]. Despite the lack of solid experimental evidence, there are theoretical hypotheses of pathophysiology for the development of glomerular damage in cancer patients, like aberrant immune activities [3]. Both solid tumors and hematological malignancies could be associated with glomerulopathy, including IgA nephropathy [4]. Currently, all reported cases of malignancy complicated by IgA nephropathy were adult patients and mainly included cancer in the kidney, lung, and gastrointestinal tract [5]. In the present study, we described a rare case of a child suffering from neuroblastoma in association with IgA nephropathy.

## Case presentation

A nine-year-old boy was admitted to the Department of Nephrology at Children’s Hospital, Zhejiang University School of Medicine, in September 2021 for palpebral edema. He had no specific medical history, and his parents were not in a consanguineous marriage. The blood pressure of this patient was normal. In view of initial blood and urine tests, he was diagnosed with nephrotic syndrome because of hypoproteinemia, hypercholesterolemia, and massive proteinuria (Table 1).

**Table 1:** Laboratory data on first admission. RBC: red blood cell; WBC: white blood cell; hsCRP: hypersensitive C-reactive protein; ESR: erythrocyte sedimentation rate; C3: complement 3; C4: complement 4; NSE: neuron-specific enolase; TB: tuberculosis.

	Results	Reference range
Urine		
Occult blood	3+	Negative
RBC (/μL)	643.2	0-13.6
Protein/creatinine ratio (mg/mg)	10.38	＜0.20
24-hour urinary protein (mg)	5195.3	＜150
Culture	Negative	Negative
Blood		
WBC (thousand/mm^3^)	9.42	4.00-12.00
Hemoglobin (g/L)	147	110-155
Platelet (thousand/mm^3^)	316	100-400
hsCRP (mg/L)	0.27	0.00-8.00
Albumin (g/L)	23.3	32.0-52.0
Urea nitrogen (mmol/L)	4.03	2.80-7.60
Creatinine (μmol/L)	40	21-65
Cholesterol (mmol/L)	7.53	3.0-5.7
ESR (mm/h)	6	0-20
C3 (g/L)	1.070	0.900-1.800
C4 (g/L)	0.156	0.100-0.400
NSE (ng/ml)	27.88	0-21.47
Viral hepatitis panel	Negative	Negative
HIV, syphilis and TB screening	Negative	Negative

**Figure 1:**
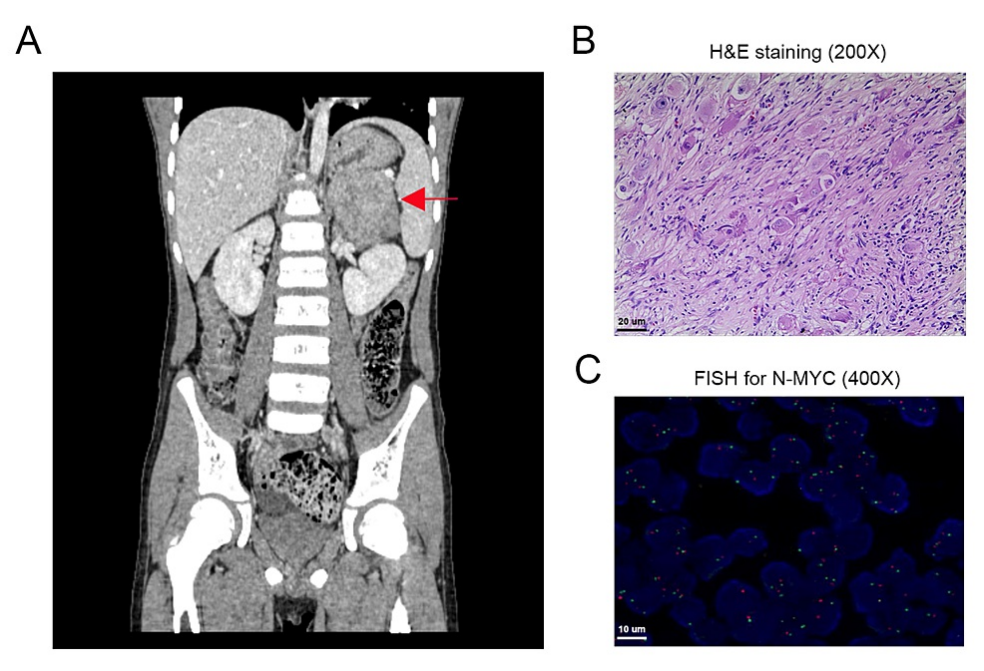
Radiographic and pathological analysis of abdominal neuroblastoma. (A) Contrast-enhanced CT images of the tumor. (B) H&E examination. (C) Fluorescence in situ hybridization for N-MYC. Red dots indicate N-MYC, green dots indicate the control gene.

Unexpectedly, abdominal ultrasonography and a contrast-enhanced CT scan revealed a tumor sizing 66mm×65mm×48mm in the left adrenal region with heterogeneous enhancement and scattered punctate calcifications, which suggests the possibility of a neurogenic tumor (Figure 1). 

There was no evidence of distant metastasis. Both kidneys were normal in size and morphology.

Then the patient was transferred to the Department of Surgical Oncology, where curative resection of the left retroperitoneal tumor and left adrenal gland was performed. The pathological analysis indicated an intermixed ganglioneuroblastoma (Figure 1). Immunohistochemical staining demonstrated that the tumor was positive for Phox2b and cytokeratin. Immunostaining for N-MYC was negative, and fluorescence in situ hybridization (FISH) also showed no amplification of N-MYC. There was no regional lymph node metastasis in all dissected nodes. These pathological results confirmed the diagnosis of neuroblastoma. The tumor was defined as a very low-risk disease based on the radiological and molecular analysis, and thus no chemotherapy or radiotherapy was given postoperatively. ﻿

**Figure 2:**
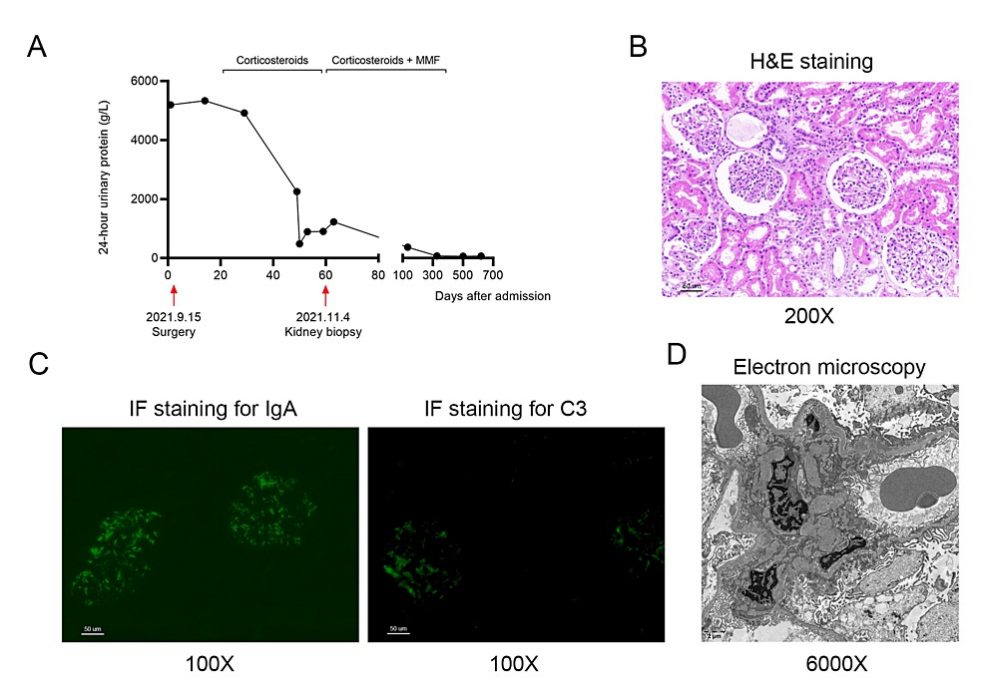
24-hour urine protein analysis and histological examination of IgA nephropathy. (A) 24-hour urine protein results over the treatment course. (B) H&E examination. (C) Immunofluorescence (IF) staining of IgA and C3. (D) Electron microscopic analysis. MMF, mycophenolate mofetil.

Two weeks after surgery, massive proteinuria remained, and the patient was thus given oral prednisone at a dose of 2 mg/kg/d to treat nephrotic syndrome. Clinical symptoms like edema and proteinuria improved significantly following treatment. However, 24-hour urine protein did not become negative and increased again after four weeks of treatment (Figure 2).

This finding suggested resistance to corticosteroids, and a renal biopsy was performed thereafter. The pathological analysis showed glomeruli with mesangial hypercellularity, increased accumulation of mesangial matrix, mild endothelial cell proliferation, and partial narrowing of capillary lumens but no glomerular crescents (Figure 2). Besides, there was a slight vacuolar and granular degeneration of tubular cells with interstitial edema. Immunofluorescence showed positive staining of IgA (3+), IgG (2+), IgM (2+), and complement C3 (2+) in the mesangial areas (Figure 2). Electron microscopy indicated that the foot processes were diffusely fused with electron-dense deposits (Figure 2). These pathological findings suggested the diagnosis of IgA nephropathy. Treatment with oral mycophenolate mofetil (MMF) of 20mg/kg/d was therefore added in combination with corticosteroids. Proteinuria largely resolved and became negative after treatment for 10 months with a gradual dosage reduction (Figure 2). Treatment was successfully discontinued with normal 24-hour urinary protein and renal function found until the last follow-up, with a duration of two years.

## Discussion

Glomerular disease concurrent with malignancy is not uncommon. While most of the patients with cancer-associated renal conditions had membranous nephropathy or minimal change disease (MCD), IgA nephropathy was rarely observed. There were a few studies from Asian and European countries reporting cancer patients complicated by IgA nephropathy, including renal cell carcinoma, lung cancer, breast cancer, and gastrointestinal malignancies, among others [5-8]. Neuroblastoma represents the most frequently diagnosed extracranial solid tumor in childhood [9]. This malignancy originates from neural crest cells and can develop at all sites along the sympathetic chain. The common sites of tumors include the adrenal gland, mediastinum, pelvic cavity, and cervical sympathetic ganglion. Patients with neuroblastoma also exhibit heterogeneous tumor biology and, thereby, distinct clinical manifestations as well as prognosis [10-12]. Only one study from Japan reported a case of neuroblastoma complicated by nephrotic syndrome [13]. In this patient, pathological analysis of the kidney biopsy merely showed minimal changes on the H&E examination, and there was a lack of data on immunostaining for IgA or other immune complex components, which thus was not sufficiently supportive of a diagnosis for IgA nephropathy. Therefore, to the best of our knowledge, our study described the first case as having concomitant neuroblastoma and pathology-proven IgA nephropathy.

The pathological connection between cancer and glomerular disease is unclear. The Buffalo/Mna rat has been used as an experimental model with spontaneous thymoma and concurrent focal segmental glomerulosclerosis, but the true link between tumor and glomerulopathy has not been established [14]. One study showed that rats bearing xenografted liver metastatic tumors without direct invasion into the kidneys had elevations of urinary protein and IgG deposits in the glomerular tufts [15]. While there was no evidence of morphological changes in light microscopy examinations, electron-dense deposits in the basement membrane and effacement of the podocyte foot process were revealed on electron microscopy analysis in this animal model. However, the molecular mechanisms underlying cancer-associated nephropathy remain elusive. It is well-recognized that tumor progression is paralleled by aberrant immune responses. Deregulation of T cell immunology and abnormal production of cytokines are believed to be associated with glomerular damage [3,16]. The release of cancer-related antigens might result in the accumulation of antigen-antibody immune complexes in the glomerulus. The development of robust experimental animal models is required to delineate the pathophysiology of tumor-related glomerulopathy.

It has been proposed that malignancy-associated glomerulopathy represents a paraneoplastic syndrome and mostly presents as a nephrotic syndrome clinically [17]. The diagnosis of paraneoplastic glomerulopathy can usually be made when there are no specific etiologies identified and clinical remission is achieved after curative treatment of the tumor. In the present case, the child was initially admitted because of typical clinical manifestations of nephrotic syndrome. Interestingly, there was no clinical improvement, including proteinuria, after the complete removal of the retroperitoneal tumor, and proteinuria did not resolve after four weeks of treatment with corticosteroids. Among the reported cases of concomitant cancer and IgA nephropathy, the majority had nephrosis recovery after curation treatment of the tumor [5,18]. It thus seems that IgA nephropathy is likely not a paraneoplastic condition of neuroblastoma in this case, and these two diseases might be a coincidence. Nevertheless, it cannot be fully excluded that there was an etiological association, given the potential pathological and molecular relevance.

In the current case, clinical remission was finally achieved after a combination therapy of corticosteroids and mycophenolate mofetil for 10 months. This suggests that standard treatment for IgA nephropathy also shows efficacy for patients with accompanying diseases like cancer. Of note, the use of corticosteroids could lead to immunosuppression and might affect tumor progression or recurrence [19], particularly in the current clinical scenario. The neuroblastoma in our study showed low-grade malignant aggressiveness based on the imaging findings and negative N-MYC amplification on the FISH test, suggesting a low risk of recurrence. In addition, specific genetic alterations might increase the risk of developing glomerular damage in neuroblastoma. However, whole-genome sequencing or whole-exome sequencing was not performed for the tumor sample or normal tissue in this case. Further studies on more patients are needed.

## Conclusions

In conclusion, concurrent malignancy and IgA nephropathy are rare, and we present a case with neuroblastoma and IgA nephropathy. Surgical removal of the tumor did not resolve proteinuria, and clinical remission of IgA nephropathy was achieved after standard therapy of corticosteroids and mycophenolate mofetil. It is difficult to confirm the potential association between these two clinical conditions, though a paraneoplastic relationship can likely be excluded.

## References

[1] Stamellou E, Seikrit C, Tang SC (2023). IgA nephropathy. Nat Rev Dis Primers.

[2] Mustonen J, Pasternack A, Helin H (1984). IgA mesangial nephropathy in neoplastic diseases. Contrib Nephrol.

[3] Bacchetta J, Juillard L, Cochat P, Droz JP (2009). Paraneoplastic glomerular diseases and malignancies. Crit Rev Oncol Hematol.

[4] Audard V, Larousserie F, Grimbert P (2006). Minimal change nephrotic syndrome and classical Hodgkin's lymphoma: report of 21 cases and review of the literature. Kidney Int.

[5] Jiang D, Zhang X, Liu J, Cui Y, Li Y, Zheng F (2018). Triple negative breast cancer and immunoglobulin A nephropathy: A case report and literature review. Oncol Lett.

[6] Mimura I, Tojo A, Kinugasa S, Uozaki H, Fujita T (2009). Renal cell carcinoma in association with IgA nephropathy in the elderly. Am J Med Sci.

[7] Lam KY, Law SY, Chan KW, Yuen MC (1998). Glomerulonephritis associated with basaloid squamous cell carcinoma of the oesophagus. A possible unusual paraneoplastic syndrome. Scand J Urol Nephrol.

[8] Yahata M, Nakaya I, Sakuma T, Sato H, Aoki S, Soma J (2013). Immunoglobulin A nephropathy with massive paramesangial deposits caused by anti-vascular endothelial growth factor therapy for metastatic rectal cancer: a case report and review of the literature. BMC Res Notes.

[9] Maris JM, Hogarty MD, Bagatell R (2007). Neuroblastoma. Lancet.

[10] Poggi GM, Fognani G, Cuzzubbo D, Liguori A, Resti M, Pela I (2011). Neuroblastoma presenting with acute kidney injury, hyponatremic-hypertensive-like syndrome and nephrotic proteinuria in a 10-month-old child. Case Rep Oncol.

[11] Maris JM (2010). Recent advances in neuroblastoma. N Engl J Med.

[12] Drukker CA, Heij HA, Wijnaendts LC, Verbeke JI, Kaspers GJ (2009). Paraneoplastic gastro-intestinal anti-Hu syndrome in neuroblastoma. Pediatr Blood Cancer.

[13] Zheng HL, Maruyama T, Matsuda S (1979). Neuroblastoma presenting with the nephrotic syndrome. J Pediatr Surg.

[14] Hirokawa K, Utsuyama M, Kasai M, Konno A, Kurashima C, Moriizumi E (1990). Age-related hyperplasia of the thymus and T-cell system in the Buffalo rat. Immunological and immunohistological studies. Virchows Arch B Cell Pathol Incl Mol Pathol.

[15] Takeda S, Chinda J, Murakami T (2012). Development of features of glomerulopathy in tumor-bearing rats: a potential model for paraneoplastic glomerulopathy. Nephrol Dial Transplant.

[16] Faria TV, Baptista MA, Burdmann EA, Cury P (2010). Glomerular deposition of immune complexes as a first manifestation of malignant melanoma - a case report. Ren Fail.

[17] Lien YH, Lai LW (2011). Pathogenesis, diagnosis and management of paraneoplastic glomerulonephritis. Nat Rev Nephrol.

[18] Kocyigit I, Dortdudak S, Eroglu E (2013). Immunoglobulin A nephropathy could be a clue for the recurrence of gastric adenocarcinoma. Nefrologia.

[19] Magyarlaki T, Kiss B, Buzogány I, Fazekas A, Sükösd F, Nagy J (1999). Renal cell carcinoma and paraneoplastic IgA nephropathy. Nephron.

